# Catechol 1,2-Dioxygenase From *Paracoccus* sp. MKU1—A Greener and Cleaner Bio-Machinery for *cis, cis*-Muconic Acid Production by Recombinant *E. coli*

**DOI:** 10.3389/fbioe.2021.703399

**Published:** 2021-11-01

**Authors:** Manikka Kubendran Aravind, Perumal Varalakshmi, Swamidoss Abraham John, Balasubramaniem Ashokkumar

**Affiliations:** ^1^ Department of Genetic Engineering, School of Biotechnology, Madurai Kamaraj University, Madurai, India; ^2^ Department of Molecular Microbiology, School of Biotechnology, Madurai Kamaraj University, Madurai, India; ^3^ Centre for Nanoscience and Nanotechnology, Department of Chemistry, Gandhigram Rural Institute, Gandhigram, India

**Keywords:** *cis, cis*-muconic acid, bioplastics, catechol 1, 2-dioxygenase, *Paracoccus* sp., recombinant *E. coli*, fed-batch culture

## Abstract

Cis, *cis*-muconic acid (ccMA) is known for its industrial importance as a precursor for the synthesis of several biopolymers. Catechol 1,2-dioxygenase (C12O) is involved in aromatic compounds catabolism and ccMA synthesis in a greener and cleaner way. This is the first study on *C12O* gene from a metabolically versatile *Paracoccus* sp. MKU1, which was cloned and expressed in *E. coli* to produce ccMA from catechol. From the *E. coli* transformant, recombinant C12O enzyme was purified and found to be a homotrimer with a subunit size of 38.6 kDa. The apparent *K*_m_ and *V*_max_ for C12O was 12.89 µM and 310.1 U.mg^−1^, respectively, evidencing high affinity to catechol than previously reported C12Os. The predicted 3D-structure of C12O from MKU1 consisted of five α-helices in N-terminus, one α-helix in C-terminus, and nine β-sheets in C-terminus. Moreover, a unique α-helix signature ‘EESIHAN’ was identified in C-terminus between 271 and 277 amino acids, however the molecular insight of conservative α-helix remains obscure. Further, fed-batch culture was employed using recombinant *E. coli* expressing *C12O* gene from *Paracoccus* sp. MKU1 to produce ccMA by whole-cells catalyzed bioconversion of catechol. With the successive supply of 120 mM catechol, the transformant produced 91.4 mM (12.99 g/L) of ccMA in 6 h with the purity of 95.7%. This single step conversion of catechol to ccMA using whole-cells reactions of recombinants did not generate any by-products in the reaction mixtures. Thus, the recombinant *E. coli* expressing high activity C12O from *Paracoccus* sp. MKU1 holds promise as a potential candidate for yielding high concentrations of ccMA at faster rates in low cost settings.

## 1 Introduction

*Cis, cis*-Muconic acid (ccMA), a C6 unsaturated dicarboxylic acid, is an industrially important precursor molecule employed for the synthesis of a broad range of economically valuable compounds of polymeric in nature (Salvachúa et al., 2018). The functionally active dual-acid structure and open benzene frame of ccMA triggers multiple chemical reactions, which lead to the formation of numerous synthetic polymers and pharmaceuticals (Vardon et al., 2016). Muconic acid can be converted into adipic acid and terephthalic acid by direct hydrogenation and Diels-Alder reaction followed by oxidation, respectively (Lu et al., 2015). The adipic acid and terephthalic acid are the major platform chemicals used primarily for the production of bioplastics and polyesters including polyethylene terephthalate and nylon-6,6. Annually 73.8 million tonnes were produced worldwide, yet the demand was increasing drastically due to its extensive applications in various industrial sector (Curran et al., 2013). Thus, various methodologies were developed for the chemical synthesis of ccMA by oxidation of petroleum derived benzene as a feedstock. However, ccMA synthesis by oxidation of benzene starts from a carcinogen and involves many hazardous and toxic chemicals. Besides, the production of an economically important chemical constituent by non-renewable sources appears miserable and such chemical methods require high energy inputs to produce ccMA, and also liberate harmful greenhouse gases into the environment (Weber et al., 2012). In this concern, the biological method seems to be a promising and eco-friendly approach for the production of ccMA. To date, many microorganisms were employed to produce ccMA through the conversion of various biomass feedstock rich in sugars and aromatic compounds (Han et al., 2015; Becker et al., 2018; Salvachúa et al., 2018). In the meantime, *Escherichia coli*—*Escherichia coli* co-culture systems were developed to achieve enhanced production of ccMA from a glucose/xylose mixture (Zhang et al., 2015). Recently, several attempts were made to develop microbial cell factories through genetic modifications, metabolic engineering by constructing artificial biosynthetic pathways, and process optimizations of metabolic pathways, which has resulted promising increase in the yield of ccMA (Xie et al., 2014; Wang et al., 2018; Choi et al., 2020). Nevertheless, such bioconversions require multiple enzyme-catalyzed reactions, the recovered yields were inadequate and their purification fold appeared to be exiguous due to other metabolic intermediates (Vardon et al., 2016). As an alternate, biosynthesis of ccMA through catechol by single enzymatic conversion using recombinant bacteria could be a superior strategy to achieve maximum production of ccMA.

Catechol is one of the most common dihydroxylated intermediates of aromatic compounds metabolism, which readily undergoes various chemical reactions due to its redox potential, results in the generation of reactive oxygen species that could harm cells and other organisms, if not eliminated from the environment (Schweigert et al., 2001). Catechol is generally metabolized through *ortho* (β-ketoadipate) and *meta* cleavage pathways, in which the intradiol cleavages are catalyzed by catechol 1,2-dioxygenase (C12O) to produce ccMA and extradiol cleavages are catalyzed by catechol 2,3-dioxygenase (C23O) to produce hydroxymuconic semialdehyde, respectively (Bugg, 2003). ccMA has been produced in a single step by the ring cleavage of catechol through the β-ketoadipate pathway by certain bacteria belonging to the genus *Pseudomonas*, *Corynebacterium*, and *Stenotrophomonas* (Guzik et al., 2013; Becker et al., 2018; Kohlstedt et al., 2018). Additionally, the crystal structures of C12O (PDB: 1DMH and 2BOY) have been determined from *Acinetobacter* sp. ADP1 and *Rhodococcus opacus* 1CP, respectively, which enabled better understanding about the interaction of the enzyme C12O with the substrate catechol (Vetting and Ohlendorf, 2000; Di Nardo et al., 2004; Matera et al., 2010). Gaining of structural information provides opportunities to redesign tailor-made enzymes with modifications in the active sites with improved catalytic activities and other related functions. Thus, the microorganisms that produce high activity C12O enzymes enable it as a suitable choice for bio-based production of high titre of ccMA in a direct single-step conversion without any by-products.

*Paracoccus* sp. MKU1 is a chemo-lithotrophic bacterium that has a unique potential to metabolize various organic compounds for its energy consumption by their peculiar nature of its genome architecture (Nisha et al., 2015). The whole-genome sequences of the *Paracoccus* sp. MKU1 substantiate the existence of three different intradiol cleavage dioxygenases such as Catechol 1,2-dioxygenase (AQY21_07,895), 6-Chlorohydroxyquinol 1,2-dioxygenase (AQY21_23,320), and Hydroxyquinol 1,2-dioxygenase (AQY21_15,640), and extradiol cleavage dioxygenases like Catechol 2,3-dioxygenase (AQY21_02,980) (Nisha et al., 2016). Moreover, the expression of gene clusters corresponding to intradiol (*C12O*) and extradiol cleavage (*C23O*) at the transcriptional levels were found to be constitutive in *Paracoccus* sp. MKU1 and their expressions were significantly induced in the presence of catechol in a concentration dependent manner (Aravind et al., 2020). The wild-type *Paracoccus* sp. MKU1 also contains genes corresponding to cis, *cis*-muconate cycloisomerase and muconolactone lyase, which could further metabolize the ccMA produced by C12O from catechol into the intermediates of the citrate cycle. Hence, it is essential to clone and overexpress *C12O* gene in a better strain heterologously, which is devoid of ccMA catabolising activity, could produce recombinant C12O enzyme in larger quantities industrially for the enhanced ccMA production. The intradiol cleavage of catechol by C12O seems to be advantageous since it is a direct single-step conversion of catechol into ccMA. Moreover, C12O catalysed conversion of catechol to ccMA looks attractive in terms of ccMA recovery with high purity even in large scale processes because it doesn’t generate any other by-products as contaminants. On the other hand, C12O efficiently catalyses the conversion of catechol in to ccMA, which is estimated to produce up to 100% molar yield of ccMA by complete utilization of catechol (Van Duuren et al., 2011). In this study, *C12O* gene from *Paracoccus* sp. MKU1 was cloned in pET30b(+) vector and transformed in to *E. coli* BL21. Initially, the biochemical properties of the purified recombinant C12O enzyme in terms of their catalytic activity and stability were characterized under different conditions prior to fed-batch fermentation. Further, the recombinant *E. coli* was employed for the fed-batch fermentation with the continuous supply of catechol as a feedstock under optimistic conditions resulted in prodigious titre of ccMA, which was successfully recovered with maximum purity.

## 2 Materials and Methods

### 2.1 Materials

4-Aminoantipyrine and Isopropyl ß-D-1-thiogalactopyranoside (IPTG) from Hi-Media, India; Catechol (extra pure, 99%) from SRL, India and ccMA from Sigma-Aldrich, India were purchased. Restriction endonuclease enzymes and T_4_ DNA Ligase enzyme were purchased from NEB, United States. pGEM-T Easy Vector Systems (Promega, United States) was used for TA cloning. *E. coli* DH5α, *E. coli* BL21 and pET30b(+) vector were procured from the original stock of our lab. Ni-NTA Agarose resin from Qiagen, Germany; Sephacryl S-300 HR resin from Cytiva, United States and NativeMark^™^ Unstained Protein Standards were obtained from Invitrogen, United States.

### 2.2 PCR Amplification of Putative C12O Gene From *Paracoccus* sp. MKU1

Genomic DNA was obtained from *Paracoccus* sp. MKU1 grown in LB medium at 37°C for 24 h using HiPurA Bacterial Genomic DNA Purification Kit (Hi-Media, India). A set of primers were designed from the genome sequences of *Paracoccus* sp. MKU1 (NCBI accession number LLWQ00000000.1) to PCR amplify full length of *C12O* gene: *C12O*F 5′-GCGGA​TCCCAT​GAC​TGT​CAA​GAT​TTT​CGA​C-3′; and *C12O*R 5′- TACTCGAGTCAGG CGGCAGCGCGAC-3′. The *C12O* gene was amplified with according to the following PCR conditions: initial denaturation for 5 min at 94°C, followed by 35 cycles of denaturation at 94°C for 45 s, annealing at 54°C for 60 s, and extension at 72°C for 2 min with a final extension of 10 min at 72°C. The PCR products were then electrophoresed and gel purified using Favorgen-PCR purification kit.

### 2.3 Heterologous Expression and Purification of C12O

The amplified products of *C12O* was cloned into pGEM-T easy vector and transformed into *E. coli* DH5α. Positive clones containing plasmids with inserts were extracted and sequence verified (SciGenom Labs, India) to confirm the presence of the *C12O* gene. The sequence-verified clones were digested using *Bam*HI and *Xho*I for release of the *C12O* gene, which was sub-cloned into pET30b(+) vector to transform into *E. coli* BL21 (DE3). Subsequently, the positive clones were sequence verified with a pair of primers from the T7 promoter region. The recombinant *E. coli* BL21 (DE3) harbouring *C12O* gene was cultured under shaking conditions at 150 rpm in LB medium (10 g/L of tryptone, 5 g/L of yeast extract, 10 g/L of NaCl; pH 7.5 ± 0.2) containing kanamycin (50 mg/L) until the culture reached an OD600 of 0.5–0.6, and the expression of the *C12O* gene was induced with IPTG (0.2–1.0 mM) by culturing at 25°C for about 3 h. Later, cells were harvested by centrifugation for 5 min at 4°C and 8,000 rpm, resuspended in lysis buffer [50 mM Tris-HCl pH 7.6; 10% glycerol; 0.1% Triton X-100; 100 μg/ml (1000 U) of lysozyme (Thermo Scientific, United States) for cell wall lysis; 1 mM PMSF as serine protease inhibitor] and sonicated at ice-cold conditions. Insoluble debris was removed by centrifugation at 12,000 rpm for 20 min at 4°C to score the supernatant containing crude enzyme. The supernatant was applied to a column packed with Ni-NTA agarose resins for purification of the recombinant enzyme with His-tag by metal-affinity chromatography, according to the manufacturer’s instructions (Qiagen, Germany). The homogeneity and molecular mass of the purified C12O enzyme was analyzed using SDS-PAGE. To determine the molecular mass of purified C12O under non-denatured conditions, Size exclusion chromatography (SEC) was performed using BioLogic DuoFlow^™^ Chromatography FPLC System. For this, sephacryl S-300 HR column was equilibrated with 50 mM Tris-HCl buffer at pH 8.5. The conditions for SEC were set at the flow rate of 1 ml/min at 9 PSI pressure and the absorbance was measured at 280 nm with QuadTec UV/Vis Detector. NativeMark^™^ Unstained Protein Standards having molecular weight in the range of 20–1,236 kDa (LC0725, Invitrogen) was used as reference protein for the calibration. The purified recombinant enzymes were stored at 20°C and used for further characterization.

### 2.4 Catechol Dioxygenase Assay

Enzymatic conversion of catechol into ccMA through intradiol cleavage mediated by the purified C12O were spectrophotometrically determined using 4-aminoantipyrine assay by measuring the reduction in the level of catechol as reported earlier (Larue et al., 1964). For this, the enzyme-substrate reactions were initiated by adding 20 µL of purified C12O enzymes with 960 µL of Tris-HCl buffer pH 7.6 containing 20 µL of 50 mM concentration of catechol, and the reaction mixture was incubated at 37°C for 20 min. After the enzyme substrate reactions, the residual catechol was measured by adding 4-aminoantipyrine and the absorbance was measured at 540 nm. The protein concentration was determined by Bradford assay. One unit of enzyme activity was defined as the amount of enzyme that required to convert 1 µmol of the catechol into ccMA per min at pH 7.5 and 37°C. Specific activity of the enzyme was defined as units per mg of protein. Each assay was performed in triplicates.

### 2.5 Effect of pH and Temperature on Activity and Stability

The optimum pH of purified recombinant C12O enzyme was determined by measuring the enzyme activity at 37°C over the pH ranges from 3.5 to 9.0, using the following buffers: 0.05 M sodium citrate (pH 3.5–4.5); 0.05 M sodium acetate (pH 5.0–6.0); 0.05 M potassium phosphate (pH 6.5–7.5); 0.05 M Tris-HCl (8.0–9.0). The optimum temperature on C12O activity was determined by recording the enzyme activity at various temperatures ranging from 15°C to 65°C. The stability of C12O at different pH/temperature was determined by incubating the enzymes at various pH conditions for 3 h at 4°C or various temperatures ranging from 10°C to 70°C for 6 h and the enzyme activity was assayed at standard conditions. The maximum stability was considered as 100% and other activities were expressed as the relative maximum activities.

### 2.6 Effect of Metal Ions on Enzyme Activity

The impact of metal ions on the catalytic conversion of catechol into ccMA by C12O was measured at standard assay conditions in the presence of trivalent and divalent metal complexes Fe^3+^, Fe^2+^, Mg^2+^, Hg^2+^, Cu^2+^, and Mn^2+^ (0.1 mM each). The reactions were carried out with 50 mM Tris-HCl buffer solution (pH 7.5) containing each of the metal ions individually at 37°C. The activity assessed in the absence of metal ions was treated as control.

### 2.7 Determination of Kinetic Constants

The kinetic parameters (*K*_m_ and *V*_max_) were determined by measuring the initial rate of enzymatic activity with different concentrations of catechol (0–200 μM) in Tris-HCl buffer solution (50 mM; pH 7.5) at 37°C for 20 min. Three independent assays were performed for each substrate concentration. The *K*_m_ and *V*_max_ values were calculated from Michaelis-Menten equation using GraphPad Prism 8 and verified with Lineweaver-Burk plots by applying the reciprocal values obtained from Michaelis-Menten equation. The *k*_cat_ value of C12O was calculated based on the molecular weight of the enzyme subunit (38.6 kDa).

### 2.8 *In silico* Analysis of C12O

The amino acid sequence of catechol 1,2-dioxygenase was retrieved from the whole genome sequence of *Paracoccus* sp. MKU1 (Accession number LLWQ00000000.1), which was subjected to PROSITE analysis to predict the active sites, substrate binding sites, and functional domains of C12O. The conservation of domains across various species was demonstrated phylogenetically by the construction of a neighbourhood joining tree with 1,000 bootstrap confidence level using MEGA 8 software. Later, the 3D structure of C12O was generated by homology modelling strategy using I-TASSER server. Concurrently, the homology modelling was validated by Ramachandran plot to assess the quality of predicted structural configuration by analysing the sterically allowed region of amino acids in the core graph. The 3D structure of ligand (catechol) was retrieved from the PubChem database and converted into PDB format using UCSF chimera. Molecular docking studies were carried out using AutoDock Tools 1.5.7 (ADT). Initially, the deletion of water, addition of polar hydrogen, merging of nonpolar hydrogen and insertion of Kollman charges were performed to pre-prepare of grid parameter file (GPF). Later, the docking parameter was outlined using the Lamarckian genetic algorithm (LMA) that comprises a maximum number of 25 × 10^5^ energy appraisals with 0.02 mutation rates and 0.8 crossover rates. The docking parameter file (DPF) was finally designated with a total 50 independent runs and programmed in the Cygwin platform. The catechol molecule docked with the 3D model of C12O was analyzed in Biovia Discovery Studio to determine the substrate binding site, amino acids interaction, the types of bond formation, and catechol cleavage upon binding with the amino acid residue of C12O enzyme in *Paracoccus* sp. MKU1.

### 2.11. Process Optimization and ccMA Production

Overnight cultures of recombinant *E. coli* BL21 transformed with pET30b(+)-*C12O* expression constructs were inoculated in the M9 modified minimal media (pH 7.5) containing glycerol (1%) as a major carbon source and 100 µM FeCl_3_. The composition of M9 modified minimal media is 1.8 g/L of K_2_HPO_4_; 1.2 g/L of KH_2_PO_4_; 4 g/L of NH_4_Cl; 0.2 g/L of MgSO4; 0.1 g/L of NaCl. The culture broth was incubated at 37°C for 6 h. Subsequently, *C12O* expression was induced with 0.2 mM IPTG and 0.2% of lactose for 6 h at 30°C. To determine the conversion of efficiency of catechol to ccMA, the induced cultures of recombinants were supplied with different concentrations of catechol ranging from 10 to 50 mM and incubated for 1 h at 35°C separately. After incubation, cultures were centrifuged and their supernatant was analyzed for the residual catechol concentration by 4-aminoantipyrine assay**.**

The fed-batch fermentation was further carried out in a 1 L shake flask supplied with 20 mM catechol at 1 h interval until 6 h under above described media and conditions. The growth of recombinant *E. coli*, media pH, reduction of catechol, and ccMA production was monitored for 6 h at 1 h interval. Metabolites in the culture supernatant were harvested, filtered through 0.22 µm filters, and analyzed for the detection of ccMA by GC-MS. GC-MS was performed with Agilent GCMS 5977B instrument using GC-capillary column (0.32 mm × 50 m fused silica) in auto injection mode with helium as the carrier gas at 100 kPa (1 bar. 14.3 psi) at the column temperature of 40°C for 2 min and raised to 250°C by an increase of 10°C/min. Further, the Mass spectrum was carried out using Agilent triple-axis detector (TAD) with electron multiplier (EM) by the scan range of 30–600 at 230°C with the electron ionization of 70 eV.

### 2.12 Purification and Recovery of ccMA

The ccMA produced in the fed-batch culture was scored by centrifugation at 6,000 rpm for 5 min to remove the bacterial biomass. The supernatant was collected in a screw cap bottle and treated with activated charcoal carbon for 1 h, which was passed through 0.22 µm filters. The flow-through liquid from 0.22 µm filtration was colorless and watery in nature, which was subjected for the acid-temperature shift (pH 2 by H_2_SO_4_ at 5°C) that allowed precipitation of ccMA. Later, the precipitate containing ccMA was dried in a vacuum oven (Spinco, India) at 50°C and washed twice with ethanol to remove the impurities if any. Finally, the quantity and purity of ccMA was analyzed by Reverse-phase High-Performance Liquid Chromatography (RP-HPLC). Shortly, RP-HPLC was performed on a Shimadzu HPLC (Prominence LC-20AT, Japan) using Suplex column (15 cm × 4.6 mm, 5 µm). The mobile phase was carried out with [A] 20 mM phosphoric acid: [B] acetonitrile; with a ratio of 75:25. The column temperature was maintained at 30°C with a flow rate of 1.5 ml/min. The sample was detected at 260 nm by diode array detector.

## 3 Results and Discussion

### 3.1 Cloning of C12O From *Paracoccus* sp. MKU1

*Paracoccus* sp. MKU1 is metabolically versatile bacteria that possess higher flexibility in utilizing wide range of recalcitrant xenobiotics (Aravind et al., 2020). Genome analysis of *Paracoccus* sp. MKU1 predicted at least 28 different putative dioxygenase genes (Nisha et al., 2016). Of which, the existence of genes corresponding to the catabolism of catechol and protocatechuate branches of the β-ketoadipate pathway has gained much significance in the biodegradation of diverse aromatic pollutants. The extradiol dioxygenase encoding *C12O* gene (AQY21_07895) corresponding to the conversion of catechol into ccMA was PCR amplified using genomic DNA as the template and gene-specific primer sets. The *C12O* gene consisted of an open reading frame (ORF) spanning about 939 bp (Supplementary Figure S1A) that encodes a protein of 312 amino acid residues with a theoretical molecular mass of 34.1 kDa and pI of 4.84. The BLAST analysis of C12O nucleotide sequence of *Paracoccus* sp. MKU1 with various closely related bacteria using mega X software revealed maximum identity with *Paracoccus pantotrophus* (90.1%) and *Paracoccus denitrificans* (89.36%), respectively. Likewise, the other bacteria genera such as *Martelella* sp. AD-3, *Rhizobium* sp. WL3 and *Labrys neptuniae* strain KNU-23 shared second-order higher homology with *C12O* gene of *Paracoccus* sp. MKU1. To our surprise, the C12O gene was chromosomally encoded in most of the bacteria except *Rhodobacter* sp. CZR27 and *Ensifer adhaerens* strain Corn53, where it was encoded in the plasmids. The PCR amplicons were cloned in pGEM-T Easy vector and transformed into *E. coli* DH5α. From the transformants, the recombinant plasmids were isolated and digested with *EcoRI* to release the insert, which confirmed the presence of *C12O* ORF (Supplementary Figure S1B). Subsequently, the pGEM-T vector carrying *C12O* ORF was digested with *BamHI* and *XhoI* to release the ORF fragment of *C12O*, which was sub-cloned in pET30b(+) expression vector (Supplementary Figure S2). The positive clones carrying a full-length coding region of *C12O* was bidirectionally sequenced and verified to contain no mutations (Supplementary Figure S3).

### 3.2 Expression and Purification of C12O From *E. coli*

**FIGURE 1:**
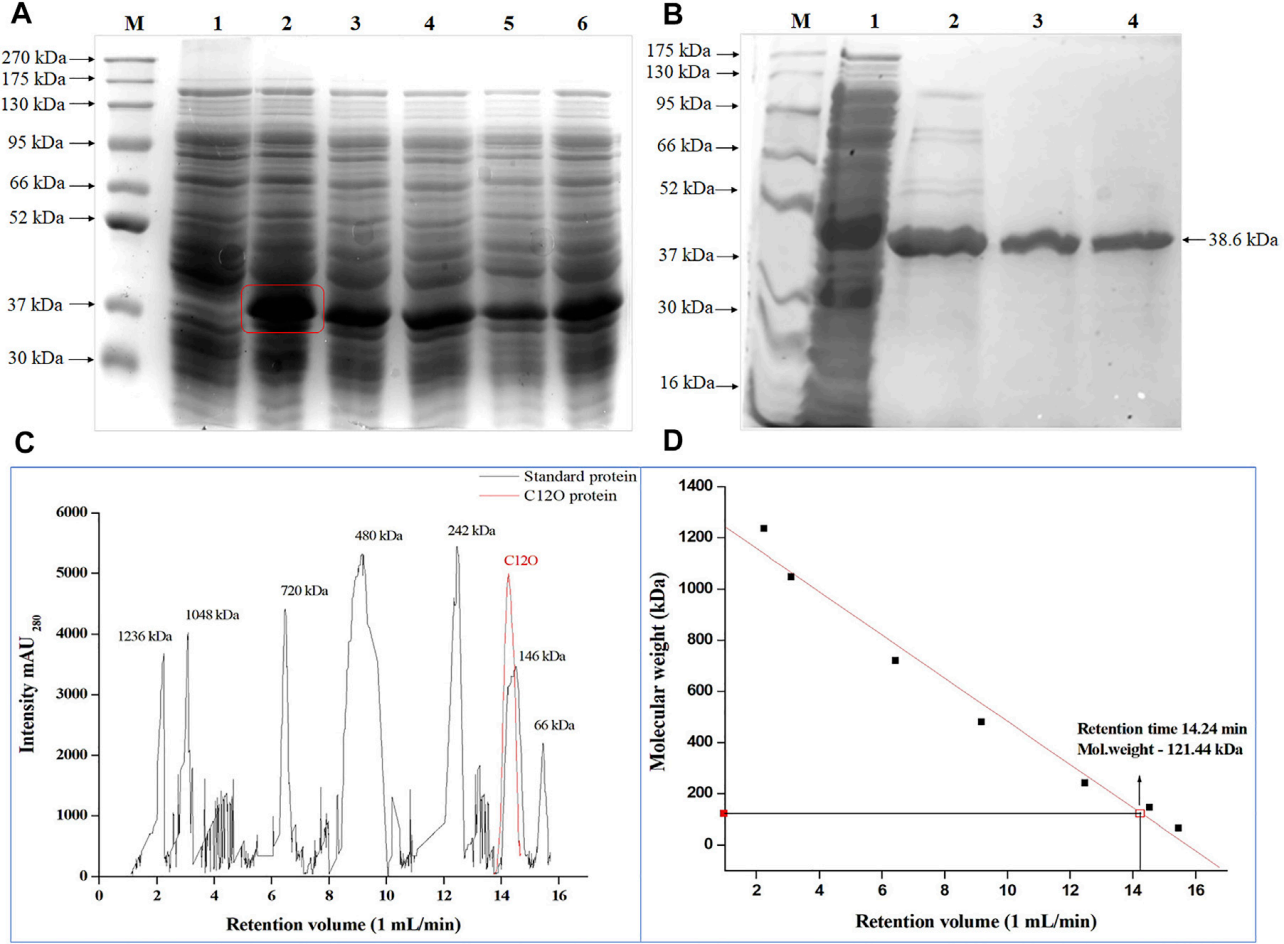
Over-expression of *C12O* from *Paracoccus* sp. MKU1 in *E. coli*. **(A)** SDS-PAGE analysis of IPTG induced cell lysate. Lane M—Protein Ladder; Lane 1—Uninduced; Lane 2—0.2 mM IPTG; Lane 3—0.4 mM IPTG; Lane 4—0.6 mM IPTG; Lane 5—0.8 mM IPTG; Lane 6—1.0 mM IPTG. **(B)** SDS-PAGE analysis of Ni-NTA column purified recombinant C12O protein after 0.2 mM IPTG induction. M—Marker, Lane 1—Crude extract, Lane 2—Eluate 1, Lane 3—Eluate 2, Lane 4—Eluate 3. **(C)**—Size exclusion chromatography analysis of purified of C12O and reference proteins. **(D)** Calibration graph for C12O.

The *C12O* ORF of *Paracoccus* sp. MKU1 cloned in pET30b(+) vector was successfully transformed into *E. coli* BL21 (DE3), and was expressed as an active protein upon induction with 0.2 mM IPTG (Figure 1A). With the 50 ml of culture, the induced expression of recombinant C12O obtained from the crude cell lysate of *E. coli* transformant was 122.48 U/ml with total activity of 6,124 Units. The recombinant C12O enzyme was purified to apparent homogeneity with a promising purification fold and recovery of 2.11 and 84.4%, respectively. The total concentration of protein in the purified fraction was 376 μg/ml. The enzyme activity of purified enzyme was decreased from 122.8 U/ml to 103 U/ml whereas, the specific activity of the purified enzyme was found to be increased from 129.9 U mg^−1^ to 274.6 U mg^−1^. The molecular mass of the purified C12O under denatured conditions on SDS-PAGE was estimated to be 38.6 kDa (Figure 1B), while the molecular mass of C12O under non-denatured conditions was determined to be 121.4 kDa by size exclusion chromatography (Figures 1C,D), suggesting that the nature of native C12O is a homotrimer. The molecular mass of C12O isolated from *Paracoccus* sp. MKU1 is similar to those of some C12Os (38.6 kDa) from *A. radioresistens* (Briganti et al., 2000; Pessione et al., 2001) but lower than those of C12O (40 kDa) from *R. rhodochrous* (Strachan et al., 1998), *A. calcoaceticus* (Patel et al., 1976) and *R. erythropolis* (Aoki et al., 1984). It should be also noted that the purified C12O from various bacteria existed mostly as dimers having two identical subunits with a molecular mass of about 29.0–38.6 kDa (Guzik et al., 2013). Exceptionally, they are present in trimeric or tetrameric structures in some bacteria including *A. radioresistens* (Briganti et al., 2000) and *R. erythropolis* (Aoki et al., 1984), respectively. Thus, C12O from *Paracoccus* sp. MKU1 is predicted to be a trimer with each monomeric unit of 38.6 kDa.

### 3.4 pH Optima and Stability

**FIGURE 2:**
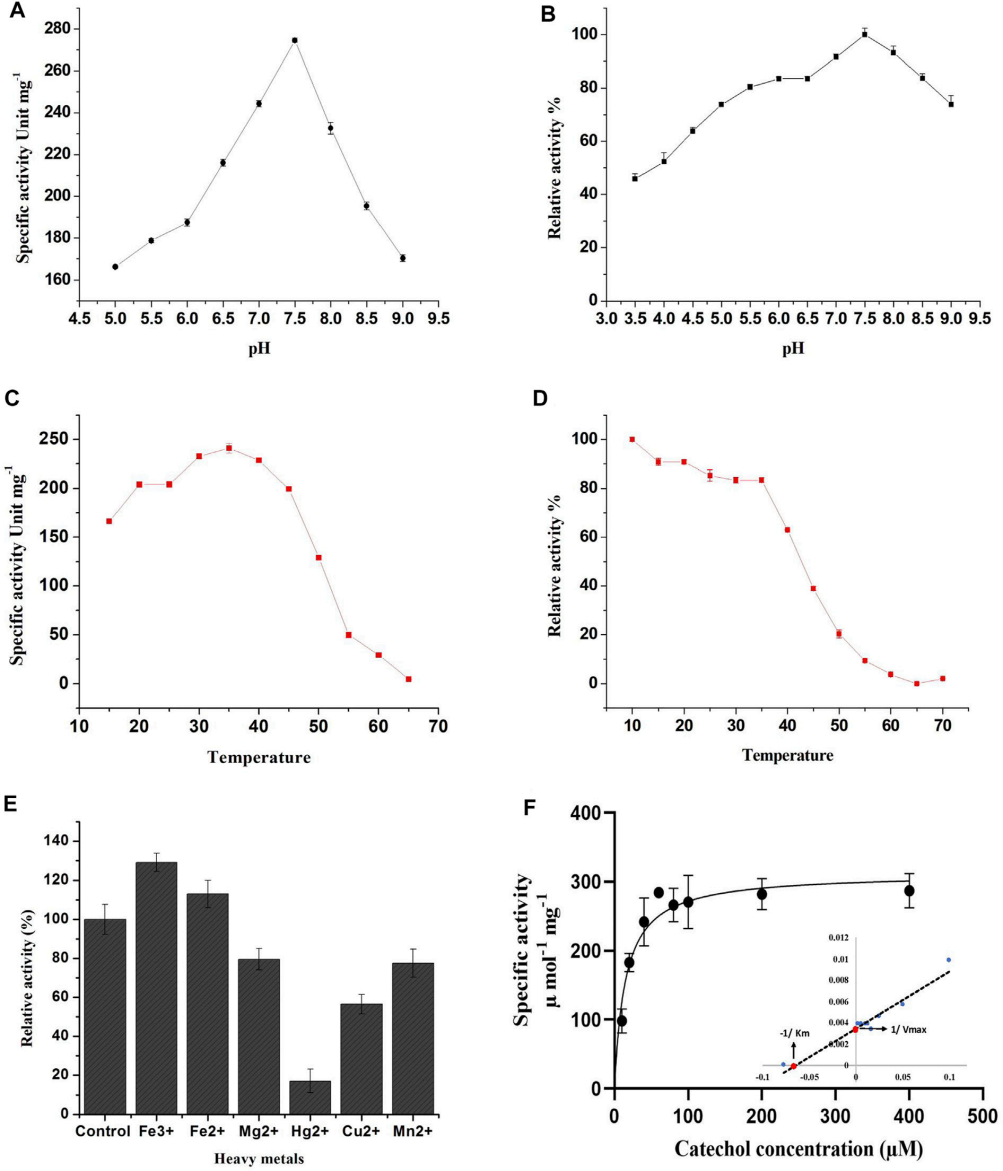
Effect of pH, temperature and metal ions on recombinant C12O activity. **(A)** pH optima, **(B)** pH stability, **(C)** Temperature optima, **(D)** Temperature stability, **(E)** Effect of metal ions, **(F)** Determination of enzyme kinetic constants. Inlet: Lineweaver-Burk plot for the determination of *K*_m_ and *V*_max_^.^ The residual relative activity of enzyme was determined by the standard assay and compared with the highest activity of the enzyme displayed at different conditions. All the assays were performed in triplicate and the values are presented as mean ± SEM.

The effect of pH on enzyme activity of the purified C12O was also investigated since pH is the crucial factor that influences spatial configuration in enzymes, which directly affects enzyme-catalyzed reactions. The purified C12O displayed a higher catalytic activity over a broad range of pH with optimum activity at pH 7.5. The C12O had maximum enzyme activity of 274.6 U mg^−1^ of protein. The enzyme activity was gradually decreased with the changes in pH towards both acidic and alkaline conditions and retained 60–65% of activity at pH 5.0 and pH 9.0 (Figure 2A). The purified C12O of MKU1 was highly active at pH 7.5 and tolerated different pH efficiently by displaying decent enzyme activity even at acidic pH of 3.5 and alkaline pH of 9.0 (Figure 2B). The reduced catalytic activity at lower branches of acidic and alkaline conditions could be attributed to the conformational changes in the C12O protein. In comparison, C12O of *Paracoccus* sp. MKU1 is more stable than C12O from other genera, as it retained 43.7% of its activity at pH 3.5. This broad range pH tolerance and stability is the unique property of this dioxygenase from *Paracoccus* sp. MKU1, since C120 purified from *P. aeruginosa* (Wang et al., 2006), *A. calcoaceticus* (Patel et al., 1976), and *Rhodococcus* sp. NCIM2891 (Nadaf and Ghosh, 2011) had shown lesser enzyme activity at acidic and alkaline pH conditions. Meantime, the pH stability of C12O was found consistent with the enzymes from *Pseudomonas* sp. AW-2 (Murakami et al., 1998) and *P. aeruginosa* (Wang et al., 2006).

### 3.5 Temperature Optima and Stability

Temperature plays a key role in maintaining the kinetic energy of an enzyme against the rate of catalytic reaction. To understand the influence of reaction temperature on enzyme activity, an assay was performed at different temperatures in the range of 15°C–65°C using catechol as substrate. The C12O enzymes exhibited maximum catalytic activity at 35°C, while enzyme activity was gradually decreased with increasing temperature (inversely proportional) and displayed less than 3% of activity at 65°C (Figure 2C). This result corroborates with observations of temperature optima for C12O from *P. putida* N6 (Guzik et al., 2011) and *A. calcoaceticus* (Patel et al., 1976). Exceptionally, thermophilic C12Os were characterized earlier from *S. xenophaga* with 50°C (Gou et al., 2009), and *Geobacillus* sp. with 60°C as temperature optima (Giedraityte and Kalėdienė, 2009).

The thermal stability of the recombinant C12O was analyzed by incubating the enzyme at different temperatures ranging from 10°C to 70°C for 3 h to evaluate the temperature stability of C12O relative to the maximum activity. The recombinant C12O enzyme stored at 10°C for 3 h poses greater stability by exhibiting maximum activity. Comparably, the enzyme stored at the temperature ranging from 15°C to 35°C had lost only 8–17% of activity. More than 81–90% of enzyme activity was lost at 50–55°C and there was complete loss of enzyme activity at 65 °C (Figure 2D). A similar effect had been previously observed for C12O from *P. aeruginosa* (Wang et al., 2006), *Arthrobacter* sp. BA-5-17 (Murakami et al., 1998) and *S. maltophilia* (Guzik et al., 2013).

### 3.6 Effect of Metal Ions on C12O Activity

The effect of different metal ions on enzyme activity of C12O was tested using catechol as a substrate (Figure 2E). The results evidenced that the presence of Fe^3+^ (FeCl_3_) and Fe^2+^ (FeSO_4_) enhanced the catalytic activity of C12O, while MgCl_2_, CuSO_4_, and MnSO_4_ slightly inhibited the activity (15–30%). HgCl_2_ was the only tested metal that strongly inhibited the catalytic activity of C12O enzyme (82.5%). Many studies evidenced previously that Hg^2+^ is an extremely effective inhibitor of the activity of C12O obtained from several microbial sources (Li et al., 2018; Setlhare et al., 2019). Moreover, the addition of Hg^2+^ (0.1 mM) resulted in complete loss of C12O activity especially in several species of *Rhodococcus* (Aoki et al., 1984; Matsumura et al., 2004; Nadaf and Ghosh, 2011) and *Arthrobacter* sp. BA-5-17 (Murakami et al., 1998). Hg^2+^ ions display high affinity towards sulfur groups of cysteine, and thus the disulfide bonds between cysteines are disrupted, which leads to structural modifications in a protein. Such modifications in the protein structure could affect the active site leading to loss of catalytic activity of enzymes. The enhanced activity by Fe^3+^ was higher than Fe^2+^ for C12O, whereas a significant increase in the presence of Fe^3+^ and Fe^2+^ had been reported previously for C12O from *Acinetobacter* sp. Y64 (Lin and Milase, 2015), *P. aeruginosa* (Wang et al., 2006), and *P. putida* (Li et al., 2018). However, the presence of Fe^3+^ and Fe^2+^ resulted in complete loss or decrease of C12O activities in *Rhodococcus* sp. (Aoki et al., 1984; Nadaf and Ghosh, 2011). C12O are metalloenzymes containing non-heme iron (Fe^3+^) as a cofactor to catalyze intradiol cleavage of catechol (Bugg, 2003). It is apparent that metal ion interactions in these enzymes play crucial role in their catalytic activity or in the structural stabilization. Replacement of Fe^3+^ and Fe^2+^ ions with other metal ions have destabilized the enzyme conformation and led to partial or a complete loss of enzyme activity (Di Nardo et al., 2004). However, the response of bacterial enzymes from various sources in the presence of heavy metals was largely varied, which indicates that the structural organizations of such enzymes from various sources might be different in response to different environmental stimulations. With respect to *Paracoccus* sp. MKU1, these findings demonstrate that none of the metal ions tested, except Hg^2+^, inhibited the activity of C12O significantly.

### 3.7 Kinetic Properties of CDOs

The Michaelis-Menten kinetics studies were determined to delineate the minimal concentration of catechol required to attain optimal catalytic cleavage by recombinant C12O enzyme. The kinetic constants for the purified C12O were determined by measuring the initial linear rates of enzymatic reaction at different concentrations of catechol (0–200 μM). The apparent *K*_m_ and *V*_max_ values for C12O were 12.89 µM and 310.1 U.mg^−1^ (Figure 2F). During kinetic characteristics determinations for C12O, substrate-level inhibition was noticed at 80 µM of catechol (Figure 2F), which corroborated with previous observations in *S. maltophilia* (Guzik et al., 2013). In comparison, the *K*_m_ of C12O was lower than a few other bacterial species (Table 1) suggesting a higher affinity of C12O to catechol than others. The *K*_m_ value of C12O from *Paracoccus* sp. MKU1 was similar to that of *S. maltophilia* and *Acinetobacter* sp. Y64 (Guzik et al., 2013; Lin and Milase, 2015), lower than in *P. chlororaphi*, *P. putida*, and *S. xenophaga* (Guzik et al., 2011; Setlhare et al., 2019) and higher than in *Rhodococcus* sp. NCIM 2891 and *Acinetobacter radioresistens* (Briganti et al., 2000; Nadaf and Ghosh, 2011). Since the *K*_m_ value of C12O from different bacterial sources varied greatly (0.19–85.19 µM), the *K*_m_ value of C12O from MKU1 signifies that the enzyme has a better affinity for catechol. Likewise, the *V*_max_ of recombinant C12O from MKU1 was several folds higher than the activity of previously reported C12O except *S. maltophilia* (Guzik et al., 2013) (Table 1). Further, the turnover number (*k*_cat_) and catalytic efficiency (*k*_cat_/*K*_m_) obtained for C12O were 199.5 s^−1^ and 1.54 x 10^7^ M^−1^ s^−1^, respectively under the optimum conditions. Previously, Rodríguez-Salazar et al. (2020) reported that the *k*_cat_ and *k*_cat_/*K*_m_ values of purified trimeric C12O from *P. stutzeri* were 16.13 s^−1^ and 1.2 × 10^6^ M^−1^ s^−1^. Likewise, *k*_cat_ values of two different isoforms of the dimeric C12O (IsoA and IsoB) from *A. radioresistens* LGM S13 were 48.7 and 31.3 s^−1^ (Briganti et al., 2000). In terms of catalytic efficiency and substrate turnover number, C12O from *Paracoccu*s sp. MKU1 reflects comparatively higher catalytic proficiency than those from *P. stutzeri, A. radioresistens and* (Briganti et al., 2000; Caglio et al., 2009; Rodríguez-Salazar et al., 2020)*.*

**TABLE 1:** Enzyme kinetic parameters K_m_ and V_max_ for Catechol 1,2-dioxygenase from various bacterial sources.

Bacterial source	K_m_ (µM)	V_max_ (U mg^−1^)	References
*Paracoccus* sp. MKU1	12.89	310.1	This study
*Pseudomonas putida*	85.19	14.54	Guzik et al. (2011)
*Pseudomonas chlororaphis*	35.76	16.67	Setlhare et al. (2019)
*Pseudomonas aeruginosa TK*U002	5.9	–	Wang et al. (2006)
*Pseudomonas putida* ND6	0.019	1.434	Zhao et al. (2007)
*Acinetobacter radioresistens*	2.82	25.8	Briganti et al. (2000)
*Rhodococcus rhodochrous*	1.1	19	Strachan et al. (1998)
*Rhodococcus opacus*	1.4	22.6	Shumkova et al. (2009)
*Rhodococcus* sp. NCIM 2891	5	62.5	Nadaf and Ghosh, (2011)
*Stenotrophomonas maltophila*	12.18	1,218.8	Guzik et al. (2013)
*Acinetobacter* sp. Y64	17.53	1.95	Lin and Milase (2015)
*Acinetobacter* sp. DS002	1.58	2	Pandeeti and Siddavattam (2011)
*Sphingomonas xenophaga* QYY	52.85	0.25 μM L^−1^ mg^−1^ min^−1^	Gou et al. (2009)
*Geobacillus* sp*.* G27	28	9.4 (k_cat_)	Giedraityte and Kalėdienė (2009)

### 3.8 Sequence Alignment, Structural and Functional Prediction of Catechol Dioxygenases

C12O from *Paracoccus* sp. MKU1 shared about 91–95% homology at the amino acid level with the sequences from other species of the same genus, besides it showed only 69–80% of homology with other genera (Supplementary Figure S4). The amino acid sequence alignment across different genera revealed the presence of a highly conserved particular stretch of sequence (101-TPRTIEGPLYVAGAP-115) in all the enzyme sequences aligned (Supplementary Figure S5). This conservation of a particular stretch of sequence at a different location between 144–158 aa had also been reported earlier (Caposio et al., 2002; Lin and Milase, 2015), where the majority of the source of enzymes were from *Acinetobacter* sp. Furthermore, it has been reported that the amino acids Ile148, Pro151, and Leu152 (highlighted in bold) in the conserved sequence encompasses the active site of C12O, and Pro151 interacts with the aromatic ring of the substrate (Vetting and Ohlendorf, 2000). The presence of a similar kind of amino acid stretch and its conservation in the C12O of *Paracoccus* sp. MKU1 demonstrated their common ancestry. The Prosite analysis for binding domains revealed the presence of stretches of amino acid sequences 136-MHGVVHGADGQPLPGAKVEVWHCDTRGFY-164, which is known as the intradiol signature of C12O enzyme that has a major role in the enzyme function such as activation and incorporation of molecular oxygen (Liang et al., 2017).

**FIGURE 3:**
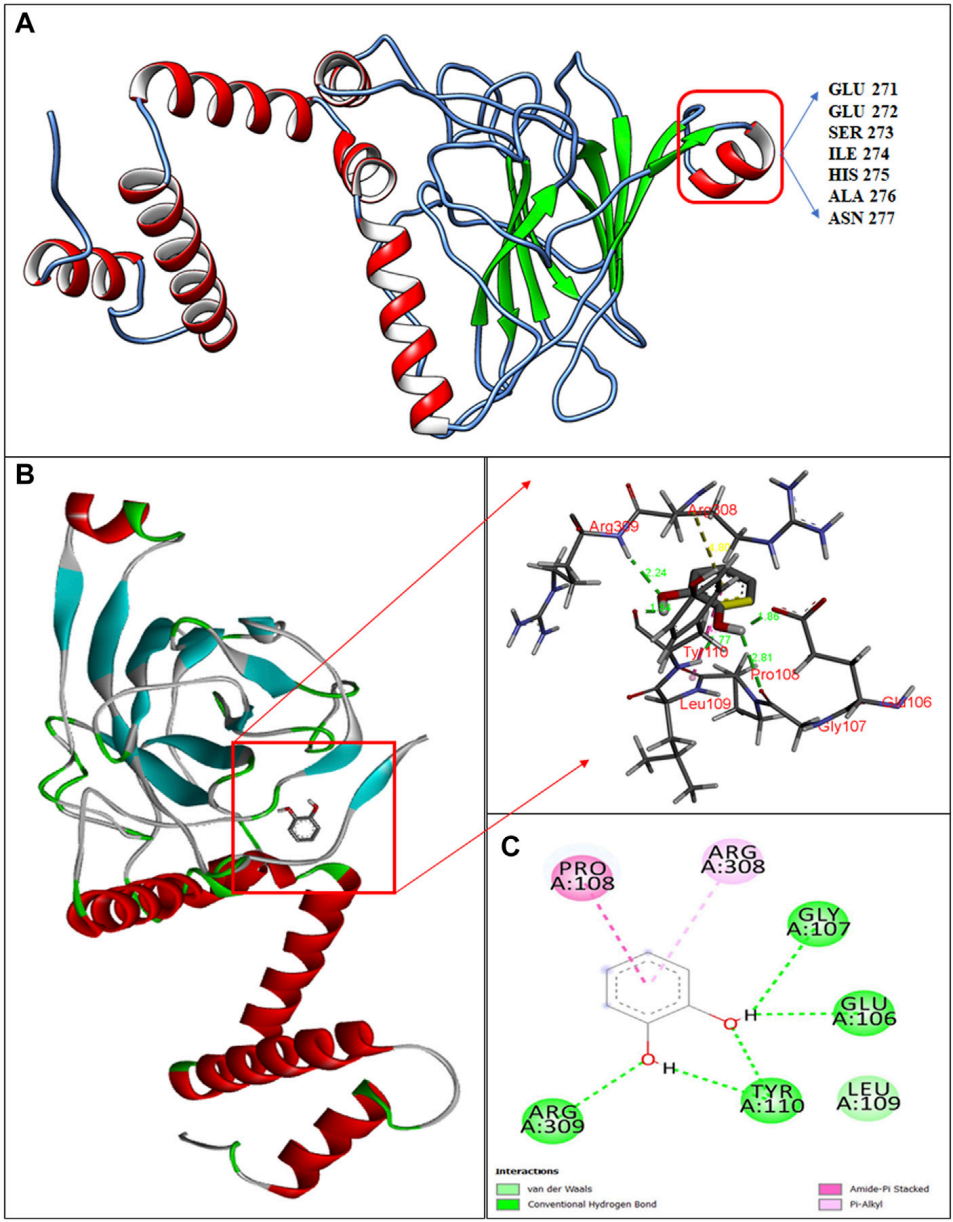
Structural insights in to catechol binding sites in C12O enzyme from *Paracoccus* sp. MKU1. **(A)** 3D structure of C12O with catechol, **(B)** Enlarged image of molecular interaction of C12O with catechol, **(C)** 2D interaction of catechol with specific amino acids of C12O enzyme.

The 3D-structure of C12O from *Paracoccus* sp. MKU1 was predicted by homology modeling using I-TASSER tool that evidenced the presence of five α-helices in the N-terminus and one α-helix in the C-terminus, whereas nine anti-parallel β-sheets appeared in the C-terminus only, which is depicted by color code using UCSF chimera as shown in Figure 3A. The quality of the homology modelling was investigated by Ramachandran plot, which evidenced 99.2% of amino acids were positioned in the favorable and core region of graph plot except GLU116 and VAL140 amino acid residues as shown in the Supplementary Figure S6. Similar molecular structures are reported previously for the C12O enzyme from several bacterial sources except for the presence of α-helix in the C-terminus (Earhart et al., 2005; Matera et al., 2010; Guzik et al., 2011). It is interesting to highlight that this unique α-helix signature in the C-terminus was located in between 271-EESIHAN-277 amino acids in C12O from *Paracoccus* and *Martelella* species only (Figure 3A; Supplementary Figure S5). The molecular insight of this conservative α-helix in the C-terminus of C12O enzyme still remains unclear. Subsequent molecular docking analysis using Auto dock tools 1.5.7 revealed that hydrogen bonding and Van-der walls interactions were formed between catechol and C12O with the binding energy of −5.07 kcal/mol. Particularly, amino acid residues Arg309, Gly107, Glu106, and Tyr110 (2 H bonds in Tyr110) were predicted to interact with the functional group of ligand molecule (catechol) through hydrogen bonds with the distance covered by the length of 2.44 Å, 2.81 Å, 2.86, and 1.84 Å and 1.77 Å, respectively (Figures 3B,C). Whereas, the Pro108 interacts with the ligand by Amide Pi stacked bond with the length of 4.17 Å and Arg308 amino acid interacts with the ligand by a pi-alkyl bond with the length of 4.80 Å (Figures 3B,C). Surprisingly, these substrate binding sites of C12O enzymes include Glu106, Gly107, Pro108, Leu109, and Tyr110 were also positioned at the conserved region in the C12Os family as shown in Supplementary Figure S5. Likewise, Han et al. (2015) had determined the presence binding pocket configured with the stretches of amino acids Leu73, Pro76, Ile105, Pro108, Leu109, Arg221, Phe253, and Ala254 facilitating the interaction between catechol and C12O from *Acinetobacter* sp. ADP1.

### 3.9 Process Optimization for Whole-Cells Catalyzed ccMA Production

**FIGURE 4:**
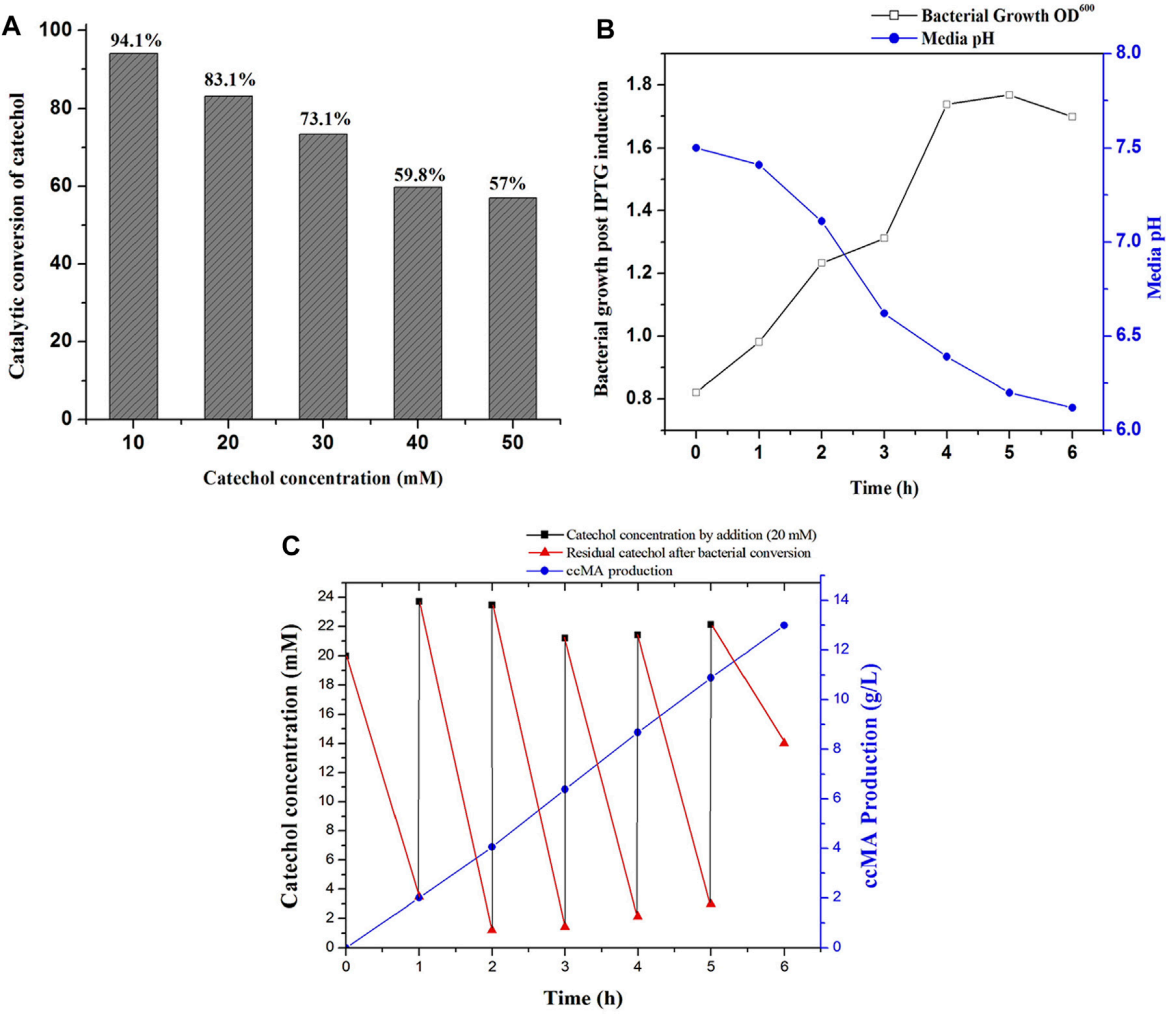
ccMA production by fed-batch fermentation. **(A)** Determination of substrate concentration for catalytic conversion by whole-cells catalyzed reactions at 1 h, **(B)** Bacterial growth profile and media pH during fed-batch fermentation, **(C)** ccMA production and catechol reduction followed by successive supply of 20 mM catechol at 1 h interval up to 6 h.

Microbial synthesis of ccMA by the IPTG induced recombinant *E. coli* expressed with *C12O* was optimized by growing them in M9 medium (100 ml) containing glycerol and different concentrations of catechol ranging from 10 to 50 mM to determine the efficiency of whole-cells catalyzed conversion of catechol to ccMA. The whole-cell catalyzed catechol conversions were performed at 35°C since the purified recombinant C12O enzyme exhibited maximum catalytic activity at 35°C only. As shown in Figure 4A, the whole-cells catalyzed conversion achieved up to 83.1% at 1 h of incubation with initial concentration of 20 mM catechol, whereas only 57.0% conversion was attained with 50 mM of initial catechol concentration at same time. Meantime, it was also confirmed that *E. coli* BL21(DE3) cells without *C12O* did not exhibit any activity towards catechol and ccMA. Likewise, Kaneko et al. (2011) reported that the whole-cells reactions by the recombinant *E. coli* expressed with *catA* gene from *P. putida* mt-2 efficiently converted 40 mM catechol in 2 h of incubation. Additionally, the culture filtrate was analyzed by GC-MS to substantiate the microbial production of ccMA by the recombinant *E. coli*, while a noticeable peak with a higher confidence level was detected at the molecular mass of 142.1 in the chromatogram, which evidenced the presence of ccMA (Supplementary Figure S7).

### 3.10 Fed-Batch Fermentation of Whole-Cells Catalyzed ccMA Production

Biosynthesis of ccMA by recombinant *E. coli* was successfully accomplished by fed-batch fermentation (1 L) with whole cells, which produces ccMA through the continuous expression of recombinant protein over a prolonged time by both IPTG and lactose autoinduction. Moreover, whole-cells catalyzed conversions do not require any additional pre-treatment like cell lysis and purification of recombinant enzymes for reactions (Kaneko et al., 2011). During fermentation, changes in the bacterial growth, media pH, catechol concentration, and ccMA production were monitored regularly at 1 h intervals until 6 h. As shown in Figure 4B, the bacterial growth was increased until 5 h of incubation and started to decline later. In the meantime, the pH of the fed-batch culture was decreased from 7.5 to 6.2, which indicated the production of ccMA as an end product of catechol conversion. The slight decrease in the pH during fermentation may inhibit the catalytic conversion efficiency, however C12O restored more than 81% of relative activity at pH 6.0–6.5 (Figure 2B). Later, cell-free extracts were harvested and processed for the recovery of ccMA, which was further analyzed by HPLC to determine its quality and quantity (Supplementary Figure S8). The productivity of ccMA was linearly increased with time and the maximum yield obtained was 12.99 g/L ccMA with 95.7% purity (Figure 4C). The quantification of ccMA synthesized during fed-batch fermentation evidenced 2.01 g/L of ccMA yield within an hour of fermentation, which confers its efficiency in producing ccMA at faster rates. The molar conversion yield of ccMA was 91.41 mM with the total successive supply of 120 mM of catechol in 6 h. The reduction in the levels of catechol was more than 90% until the 5th supply, which was directly proportional to ccMA production. However, at the 6th supply, only ∼30% of catechol was reduced and the remaining ∼70% was retained in the fermentation media over an hour (Figure 4C), and this reduced conversion of catechol might be due to the inhibitory effects on the growth of culture by the prevailing concentrations of ccMA produced and decrease in the media pH. Previously, Kaneko et al. (2011) produced 59.0 g/L ccMA in 12 h with a continuous supply of 480 mM (10 mM for 48 times at 15 min interval) catechol in a fed-batch process using a genetically engineered *E. coli* expressed with *C12O* (*catA*) gene from *P. putida* mt-2. Likewise, *K. pneumoniae* was genetically modified by deleting *catB* gene and overexpressed *C12O* that produced 2.1 g/L of ccMA from catechol endogenously (Jung et al., 2015). Lee et al. (2018) had reported the yield of 34 g/L and 54 g/L of ccMA from 7 to 50 L of the feed stock culture using a genetically engineered *C. glutamicum*, which was heterologously expressed with foreign protocatechuate decarboxylase followed by the deletion of *AroE*, *pcaG/H* and, *catB* genes for the accumulation of ccMA. ccMA was produced from vanillic acid and 4-hydroxybenzoic acid (lignin derivatives) by genetically engineered *P. putida* IDPC, which yielded 22.9–31.4 mg/L/h of ccMA (Sonoki et al., 2018). Choi et al. (2019), constructed a recombinant *E. coli* expressing a cluster of gene from different bacterial sources (*asbF* from *B. thuringiensis*; *aroY* from *K. pneumoniae*, and *catA* from *A. calcoaceticus*) for the conversion of 3-dehydroshikimate to ccMA in the fed batch fermentation, which yielded 64 g/L of ccMA with accumulation of other intermediates. Though, ccMA has been produced using certain microbes previously by metabolizing aromatic compounds including phenol, PCA and toluene, benzoate, and vanillic acid, bioconversion of catechol to ccMA in a single step by the whole-cell catalytic process using recombinant bacteria expressing high activity C12O seems to be advantageous since no other contaminants accumulated as a by-product in the fermented culture broth. Thus, the recombinant *E. coli* expressing high activity C12O from *Paracoccus* sp. MKU1 looks as a promising candidate for producing contaminant-free ccMA in higher concentrations within shorter duration and under low-cost conditions through continuous batch fermentation. However, the use of recombinant bacteria for continuous batch fermentation is not commonly employed in the industries due to the difficulties that include instability of the recombinant proteins in the host cells, risk of genetic drift in the culture, lack of process optimization, and uncharacteristic intracellular fluxes (Peebo and Neubauer, 2018). This study has successfully demonstrated the possibility of optimization of recombinant protein expression, characterization enzyme activity and stability, efficiency of catalytic conversion, determination of substrate levels, and its saturation in the fed-batch fermentation itself. It is evident from these observations that the strategies employed for the ccMA production seems logical to transfer the technology from fed-batch to continuous batch fermentation using recombinant *E. coli* and would also be a remarkable choice for the industrial operations for large scale productions.

## 4 Conclusion

In this work, we have generated a genetically stable *E. coli* transformant carrying high activity C12O gene from a metabolically versatile *Paracoccus* sp. MKU1, which is capable of producing ccMA from catechol. The purified recombinant C12O displayed high catalytic efficiency towards catechol, reasonable stability at various pH and tempearture, and excellent kinetic properties that makes it as a suitable microbial cell factory for the production of ccMA. The whole-cells catalyzed reactions (1 L) with the recombinant *E. coli* has yielded 12.99 g/L of ccMA with 95.7% purity from 120 mM of catechol in 6 h of incubation. Bio-based ccMA production using various other substrates with genetically modified organisms is regarded promising with higher yield, but the pre-treatment strategies and recovery of ccMA is a bottleneck to achieve the anticipated purity due to the existence of numerous metabolic intermediates. This direct one step enzymatic conversion of catechol to ccMA in a shorter duration without any contaminating by-products would be a better choice for many industrial applications. Though various chemical and biological methods were inspected for ccMA synthesis, still there is a great deal for constructing an optimistic strategy to synthesize ccMA to overcome its demand, especially while producing through the continuous batch fermentation. Thus, the recombinant *E. coli* transformant carrying C12O from *Paracoccus* sp. MKU1 seems to be a systemic tool with excellent flexibility for various parameters, and could be employed for the synthesis of ccMA in a greener and cleaner way by continuous fed-batch fermentation in large scale**.**

## Data Availability

The datasets presented in this study can be found in online repositories. The names of the repository/repositories and accession number(s) can be found in the article/Supplementary Material.
