# Supplementary material for: Catechol 1,2-Dioxygenase From Paracoccus sp. MKU1—A Greener and Cleaner Bio-Machinery for cis, cis-Muconic Acid Production by Recombinant E. coli
Source: Front Bioeng Biotechnol. 2021 Nov 1;9:703399. doi: 10.3389/fbioe.2021.703399 (PMC8591083; doi:10.3389/fbioe.2021.703399)
Supplement: Supplementary file 1 [file DataSheet1.DOCX]

Supplementary figures

**Supplementary Fig. 1. (A)** PCR amplification of catechol 1,2-dioxygenase gene from *Paracoccus* sp. MKU1; 1^st^ Lane – Marker; 2^nd^ Lane – *C12O* gene. **(B)** Clone confirmation by restriction digestion of pGEM-T easy vector with *EcoRI*; 1^st^ Lane - 1 Kb Ladder; 2^nd^ Lane - undigested *C12O* clone; 3^rd^ Lane - *EcoRI* digested *C12O* clone.


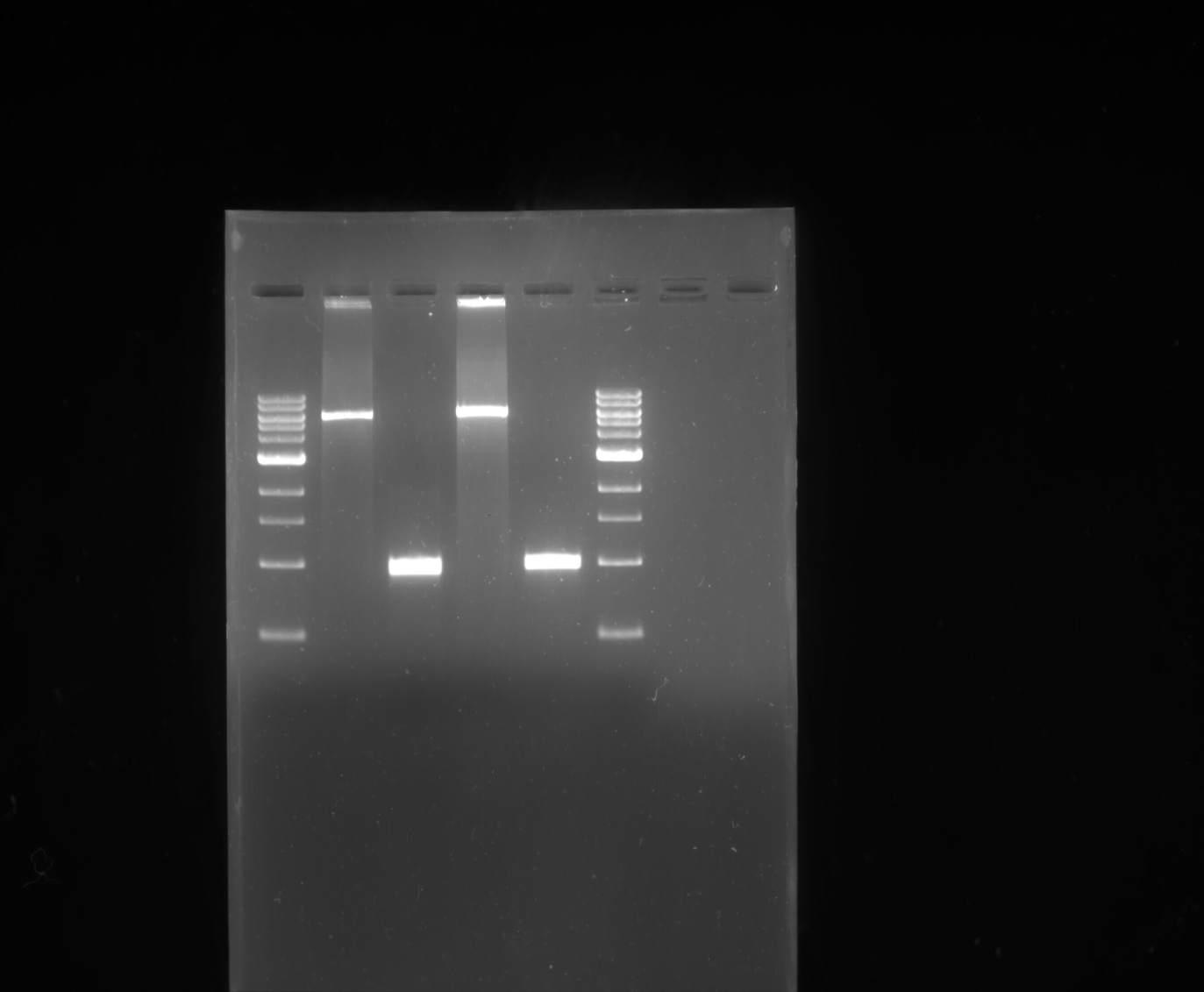


1 2 3

1.5 Kb

1.0 Kb

3.0 Kb

6.0 Kb

0.5 Kb

Digested pET30 b(+) vector

Digested C12O gene

**Supplementary Fig. 2.** Double digestion of vector and insert prior to ligation into pET30b(+) vector. 1^st^ Lane - 1 Kb marker; 2^nd^ Lane - pET30b(+) vector digested with *BamHI* & *XhoI*; 3^rd^ Lane - *C12O* amplicon digested with *BamHI* & *XhoI*.

**Supplementary Fig. 3.** Sequence verification of recombinant *C12O* in pET30b(+) vector construct. **(A) –** Nucleotide sequence of recombinant *C12O* in pET30b(+) vector; The underlined bold sequence are restriction sites; Start and stop codon were mentioned in red box; the poly-histidine sequence indicated in green box; **(B) –** Alignment score of blast results; **(C) –** Query match with NCBI database.

**Supplementary Fig. 4.** Neighbor joining tree construction by 1000 bootstrap value based on *C12O* nucleotide sequences.

**Supplementary Fig. 5.** Multiple sequence alignment of C12O based on amino acid sequences; Inlet: Conserved amino acid sequences among different genus and species are highlighted.


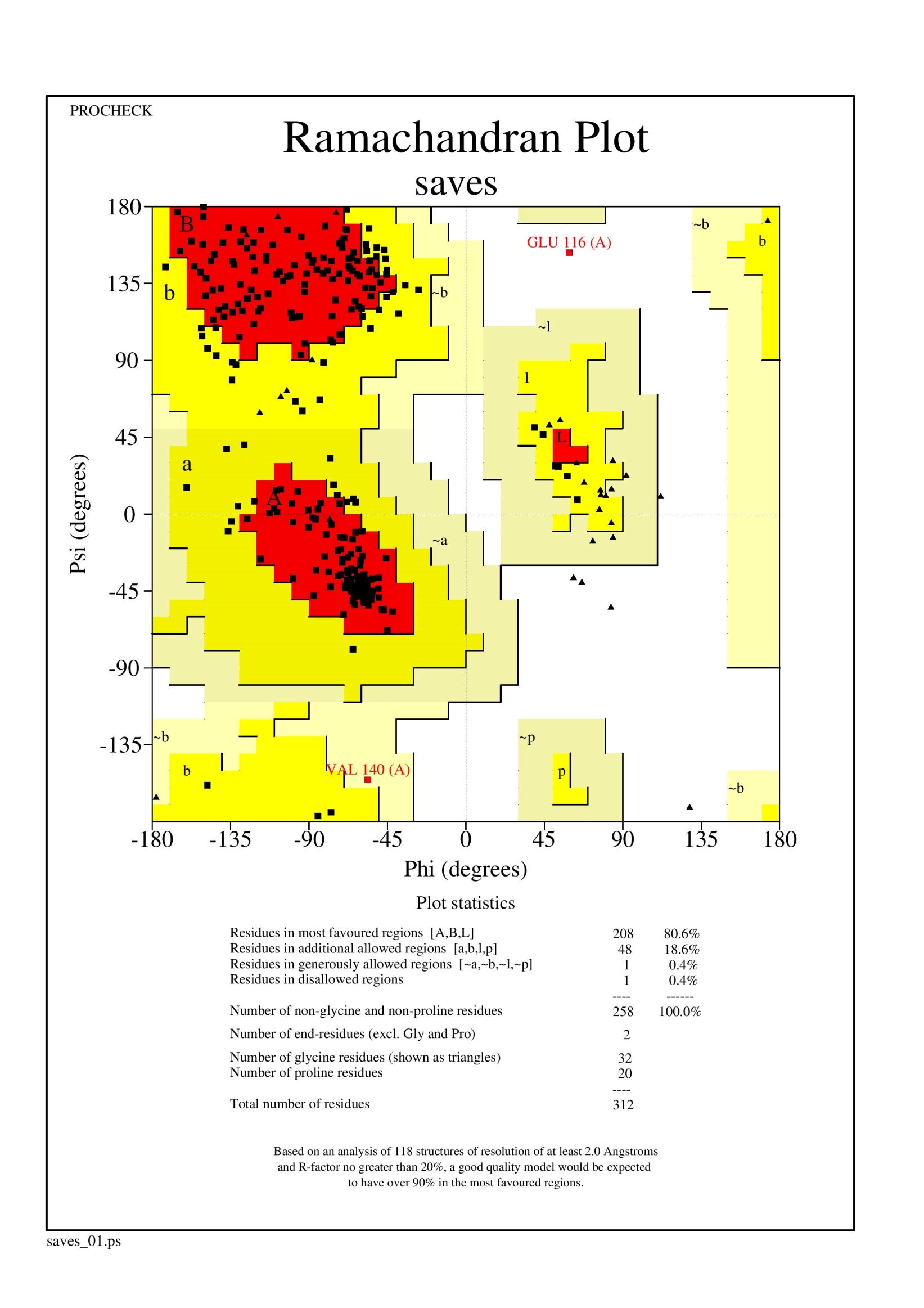


**Supplementary Fig. 6.** Ramachandran plot of Catechol 1,2-dioxygenase protein structure generated by homology modelling using I-TASSER server.

A

B


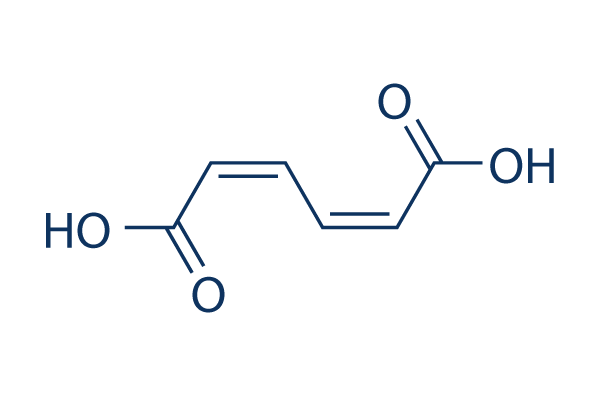


Mol. mass 142.11


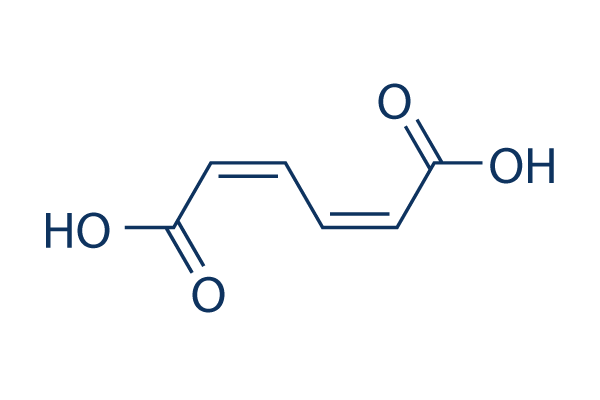


Mol. Mass 142.11

**Supplementary Fig. 7.** GC-MS analysis of culture filtrate grown with *E. coli* expressing C12O from *Paracoccus* sp. MKU1. (A) – GC-MS chromatogram; (B) – Mass spectrum.

**Supplementary Fig. 8**. Qualitative and quantitative determination of ccMA recovered from fed-batch culture by HPLC analysis. **(A)** - Standard ccMA; **(B)** Purified ccMA.
